# Adherence to HAART: A Systematic Review of Developed and Developing Nation Patient-Reported Barriers and Facilitators

**DOI:** 10.1371/journal.pmed.0030438

**Published:** 2006-11-21

**Authors:** Edward J Mills, Jean B Nachega, David R Bangsberg, Sonal Singh, Beth Rachlis, Ping Wu, Kumanan Wilson, Iain Buchan, Christopher J Gill, Curtis Cooper

**Affiliations:** 1 Centre for International Health and Human Rights Studies, Toronto, Ontario, Canada; 2 Department of International Health, Johns Hopkins Bloomberg School of Public Health, Baltimore, Maryland, United States of America; 3 San Francisco General Hospital, AIDS Division, University of California San Francisco, San Francisco, California, United States of America; 4 BC Centre for Excellence in HIV/AIDS, University of British Columbia, Vancouver, British Columbia, Canada; 5 Canadian College of Naturopathic Medicine, North York, Ontario, Canada; 6 Department of Medicine, University of Toronto, Toronto, Ontario, Canada; 7 Evidence for Population Health Unit, University of Manchester, Manchester, United Kingdom; 8 Center for International Health and Development, Boston University School of Public Health, Boston, Massachusetts, United States of America; 9 Division of Infectious Diseases, Department of Medicine, University of Ottawa, Ottawa, Ontario, Canada; University of Ottawa, Ottawa, Ontario, Canada

## Abstract

**Background:**

Adherence to highly active antiretroviral therapy (HAART) medication is the greatest patient-enabled predictor of treatment success and mortality for those who have access to drugs. We systematically reviewed the literature to determine patient-reported barriers and facilitators to adhering to antiretroviral therapy.

**Methods and Findings:**

We examined both developed and developing nations. We searched the following databases: AMED (inception to June 2005), Campbell Collaboration (inception to June 2005), CinAhl (inception to June 2005), Cochrane Library (inception to June 2005), Embase (inception to June 2005), ERIC (inception to June 2005), MedLine (inception to June 2005), and NHS EED (inception to June 2005). We retrieved studies conducted in both developed and developing nation settings that examined barriers and facilitators addressing adherence. Both qualitative and quantitative studies were included. We independently, in duplicate, extracted data reported in qualitative studies addressing adherence. We then examined all quantitative studies addressing barriers and facilitators noted from the qualitative studies. In order to place the findings of the qualitative studies in a generalizable context, we meta-analyzed the surveys to determine a best estimate of the overall prevalence of issues. We included 37 qualitative studies and 47 studies using a quantitative methodology (surveys). Seventy-two studies (35 qualitative) were conducted in developed nations, while the remaining 12 (two qualitative) were conducted in developing nations. Important barriers reported in both economic settings included fear of disclosure, concomitant substance abuse, forgetfulness, suspicions of treatment, regimens that are too complicated, number of pills required, decreased quality of life, work and family responsibilities, falling asleep, and access to medication. Important facilitators reported by patients in developed nation settings included having a sense of self-worth, seeing positive effects of antiretrovirals, accepting their seropositivity, understanding the need for strict adherence, making use of reminder tools, and having a simple regimen. Among 37 separate meta-analyses examining the generalizability of these findings, we found large heterogeneity.

**Conclusions:**

We found that important barriers to adherence are consistent across multiple settings and countries. Research is urgently needed to determine patient-important factors for adherence in developing world settings. Clinicians should use this information to engage in open discussion with patients to promote adherence and identify barriers and facilitators within their own populations.

## Abstract 2

An analysis of qualitative and quantitative studies found consistent barriers to adherence to HIV therapy across multiple settings and countries, ranging from access to medication to problems with complicated regimens.

## Editors' Summary

**Background.:**

The World Health Organization has estimated that in 2005, about 38 million people worldwide were living with HIV/AIDS; the mortality caused by HIV/AIDS is very high. Antiretroviral drugs are effective at controlling the disease and extending life span. However, it is important for people to stick to the drug regimens exactly in order to keep levels of HIV low, prevent it from becoming resistant to drugs, and stop the illness from progressing. However, many people find it very difficult to take antiretroviral drugs precisely as they should. There is already some evidence from research studies on the reasons why this is the case. There are two different research approaches taken by these studies: “qualitative” methods, which try to find out about attitudes and behaviors using focus groups, interviews, or other techniques; and “quantitative” methods, which try to find out about peoples' opinions and experience using surveys with set questions for the participants to answer, and then count the different responses.

**Why Was This Study Done?:**

The investigators wanted to put together all of the available evidence from published research studies (called doing a “systematic review”) on which factors affected people's adherence to antiretroviral drugs. They wanted to do a systematic review because it is thought to be a very rigorous way of appraising all the available evidence (although there is considerable debate about the value of using such a method to analyze the results of qualitative research).

**What Did the Researchers Do and Find?:**

The study team searched biomedical literature databases as well as conference abstracts and research registries using a defined set of search queries. They screened all the scientific papers they found; those reporting results of original research into factors affecting antiretroviral adherence were then analyzed in more detail. 84 relevant studies were identified, of which 37 used “qualitative” methods (focus groups, interviews, open-ended questioning) and 47 used “quantitative” methods (surveys). Most of these studies had been carried out in the developed world. Then, the researchers extracted the factors affecting adherence from the original studies, which could be either “positive” factors (helping adherence) or “negative” ones (making adherence more difficult). They classified the factors into four key themes: “patient related” (e.g., seeing positive results, fear of disclosure, being depressed); “beliefs about medication” (e.g., faith in how well the drugs worked, side effects); “daily schedules” (e.g., using reminder tools, disruptions to routine); and “interpersonal relationships” (e.g., trusting relations with health-care provider; social isolation).

Many barriers to adherence were common to both developed and developing settings. Some factors were unique to the studies conducted in the developing world, such as financial constraints and problems with traveling to get access to treatment. Fear of disclosure was an important barrier identified in many of the studies.

**What Do These Findings Mean?:**

The researchers combined the results of many different studies and identified factors that help or obstruct adherence to antiretroviral treatment. By identifying influences common to the different settings, greater weight can be placed on the factors that were identified. Only 12 of the studies included in this research were from the developing world, where the majority of HIV/AIDS patients live; hence more work is needed to examine and address the factors influencing antiretroviral adherence in these parts of the world. This study provides researchers and health policy makers with a starting point for changes that might help to ensure greater adherence to antiretroviral treatment.

**Additional Information.:**

Please access these Web sites via the online version of this summary at http://dx.doi.org/10.1371/journal.pmed.0030438.

- Medline Plus information on AIDS medicines (Medline Plus is a service of the US National Library of Medicine and the National Institutes of Health)
- Joint United Nations Programme on HIV/AIDS has information about the state of the HIV/AIDS epidemic worldwide
- The World Health Organization has an HIV/AIDS program site providing comprehensive information on the HIV/AIDS epidemic worldwide
- The World Health Organization pages on antiretroviral therapy

## Introduction

The introduction of antiretrovirals has been credited with extending the life span of people living with HIV/AIDS [1]. However, treatment efficacy relies on access to treatment and excellent adherence, which has proven to be a serious challenge to those receiving highly active antiretroviral therapy (HAART) [2,3]. The regimens are often complicated, can require dietary restrictions, and may lead to adverse effects [4]. Non-adherence to antiretroviral therapy in adult populations has been shown to range from 33%–88%, depending on how adherence is defined and evaluated [5]. Research indicates that consistently high levels of adherence are necessary for reliable viral suppression [6,7] and prevention of resistance [8], disease progression [9], and death [10]. As successful HIV treatment requires exceptional adherence to antiretroviral therapy, interventions to improve and maintain adherence are needed.

Several studies have been conducted that examine factors affecting adherence to HAART. We used a novel methodology to synthesize the information from these studies by performing a systematic review on all the literature available in this field using content analysis, particularly focusing on the currently existing qualitative studies and examining their generalizability through quantitative data. We examined both developed and developing nation patient populations [11].

## Methods

### Search Strategy

We performed a systematic, all-language literature search for all qualitative studies and quantitative surveys that addressed barriers and motivators influencing adherence to antiretroviral regimens in HIV-positive individuals.

We (EJM and BR) searched the following databases: AMED (inception to June 2005), Campbell Collaboration (inception to June 2005), CinAhl (inception to June 2005), Cochrane Library (inception to June 2005), Embase (inception to June 2005), ERIC (inception to June 2005), MedLine (inception to June 2005), and NHS EED (inception to June 2005). Unpublished studies were also sought using the search terms “adherence” and “HIV” on Clinicaltrials.gov, the UK National Research Register, and conference abstracts from international conference Web sites: International AIDS Society conferences (inception to 2005) and Conferences on Retroviruses and Opportunitistic Infections (inception to 2005). Our search strategy combined terms that represented attitudes, barriers, and anxieties. Our search vocabulary included “HIV” or “AIDS”, “compliance OR adherence”, “factors OR determinant* OR barriers”, “motivate* OR facilit*”, and “HAART OR antiretroviral*”. The detailed search strategy is available from the corresponding author upon request. We supplemented this search by reviewing the bibliographies of key papers.

### Study Selection

Two members of the study team (BR and PW) independently reviewed the abstracts. Eligible studies met the following criteria: (1) reported an original research study, (2) contained content addressing barriers or facilitators to antiretroviral adherence, and (3) were either a qualitative study or quantitative survey. The studies were divided to represent developed or developing nations, as according to the United Nations Human Development Index (HDI) [12]. The HDI is a composite index that measures a country's average achievements in three basic aspects of human development: longevity, knowledge, and a decent standard of living.

**Figure 1:**
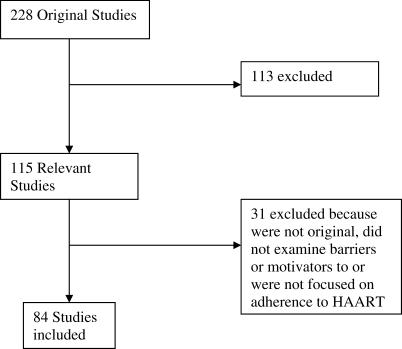
Flow Chart of Studies Included in Review

### Data Extraction

Two reviewers (BR and PW) independently extracted data and appraised both quality and content. From an initial review of qualitative studies by BR and PW, a coding template was iteratively developed to categorize key barriers to adherence to HAART. The reviewers then conducted a second review of the papers and identified whether they contained the barriers present in the complete template. At each stage of the data abstraction, the reviewers discussed the studies to determine consensus regarding the identification and coding of themes. We analyzed the themes presented in the qualitative studies. After the initial viewing of the selected articles, these themes were grouped into categories. Barriers/facilitators fell under the following subheadings: (1) patient-related, (2) beliefs about medication, (3) daily schedules, and (4) interpersonal factors/relationships. To determine the extent to which these themes exist in the wider communities of developed and developing nations, the reviewers then abstracted data from the survey studies to determine if the issues addressed in the qualitative studies had been asked about in the surveys. We abstracted data on the prevalence of the issues as reported in the surveys.

We extracted data on the quality of both qualitative and quantitative studies using pre-determined criteria for quality. We previously reported our rationale for assessing the quality of qualitative studies and in this study have extended our quality assessment to examine quantitative surveys [13]. Although no formal criteria exist for appraising the quality of surveys, we a priori determined that the following criteria are important across surveys: 1) the survey included members of the target community in the preparation of the survey tool, 2) the survey instrument was assessed for face validity, 3) the survey population was randomly selected, 4) a rationale for determining the response rate was provided, and 4) the investigators attempted to contact non-responders. We did not propose a cut-off score for higher-quality surveys versus lower-quality surveys.

**Table 1:**
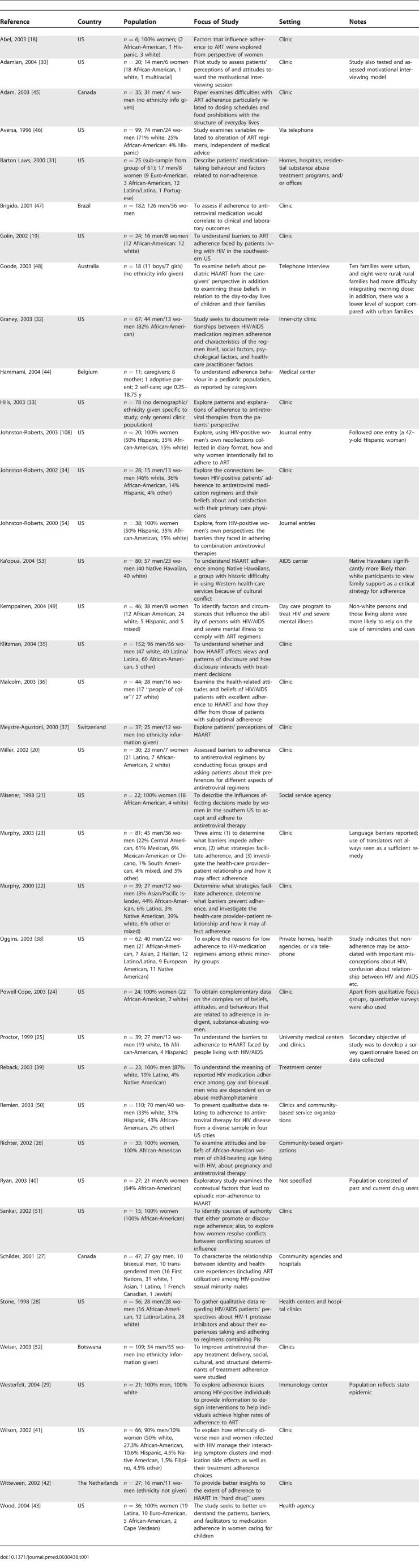
Study Characteristics

**Table 2:**
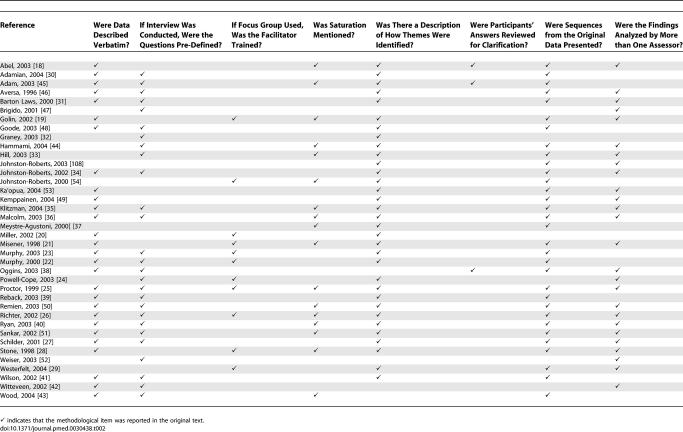
Reporting Criteria of Qualitative Studies

**Table 3:**
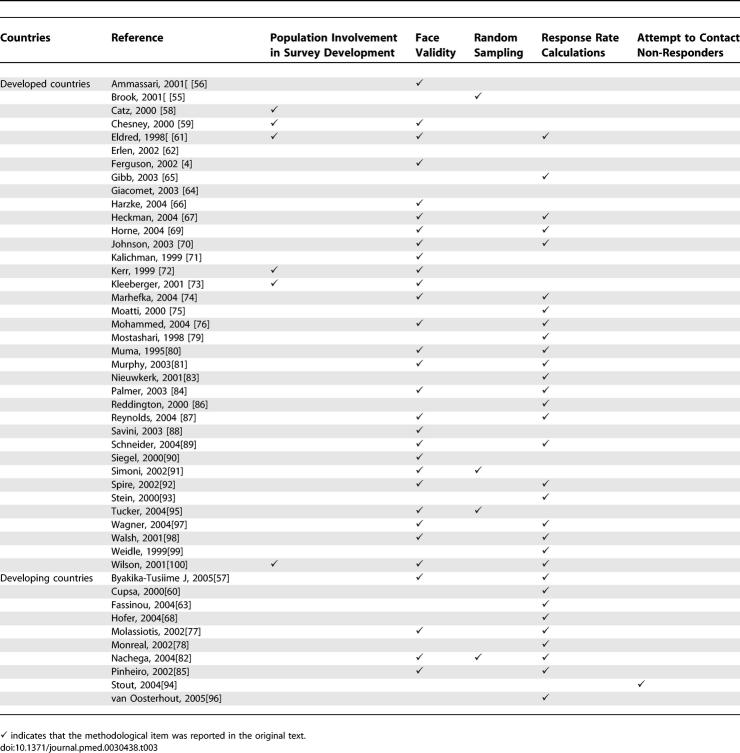
Quality Criteria for Survey Studies

### Statistical Analysis

We measured chance-adjusted inter-rater agreement for eligibility using the κ statistic. EM and PW conducted all statistical analyses. When information on proportions was available in the quantitative studies, we first stabilized the variances of the raw proportions (r/n) using a Freeman-Tukey-type arcsine square-root transformation [14], and then conducted weighted analysis of studies using methods described by Fleiss [15]. The pooled proportion is calculated as the back-transform of the weighted mean of the transformed proportions, using inverse arcsine variance weights for the fixed-effects model and DerSimonian-Laird weights for the random-effects model. The random-effects model recognizes that the studies are a sample of all potential studies and incorporates an additional between-study component to the estimate of variability [16]. Thus, larger studies with smaller variances have relatively more impact on the final estimate. We present the weighted mean with 95% confidence intervals, with lower confidence intervals truncated at zero. The I^2^ statistic was calculated as a measure of the proportion of the overall variation in the meta-analyses that was attributable to between-study heterogeneity [17].

**Table 4:**
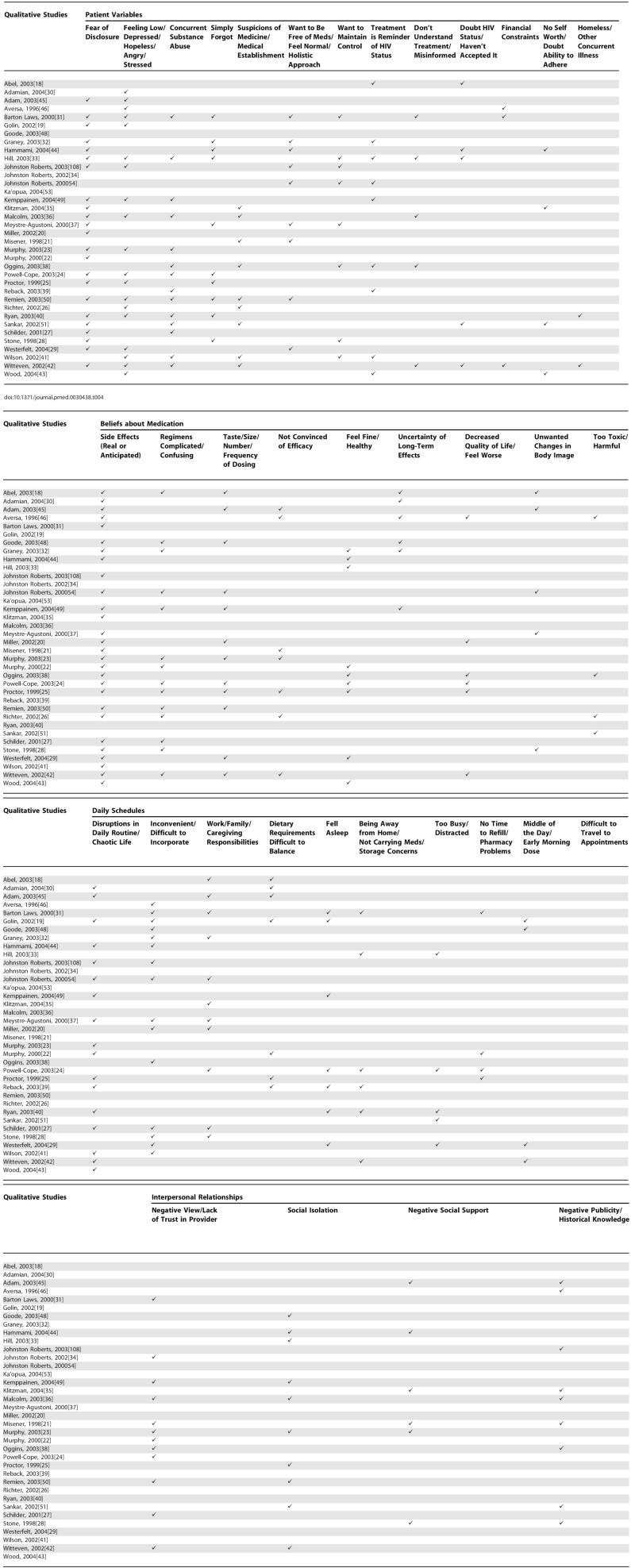
Barriers to Adherence Identified in Qualitative Studies (Developed Countries)

**Figure 2:**
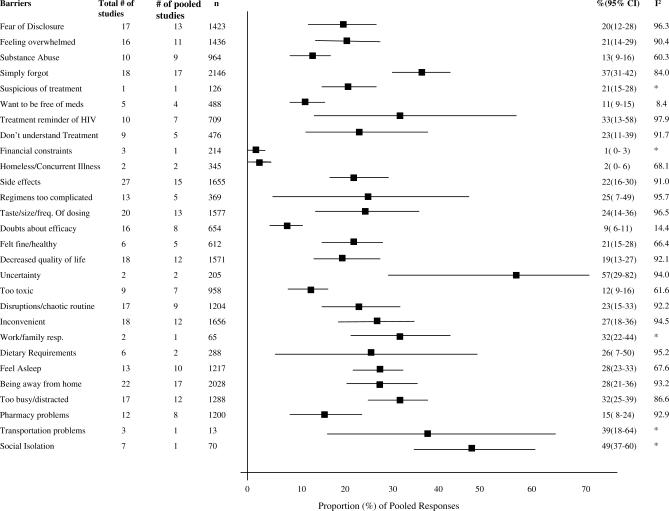
Barriers Reported in Developed Countries

## Results

### Study Selection and Characteristics

The primary literature search produced 228 studies. There was near-perfect agreement between EJM and BR on choosing the 115 applicable studies from the reviewed abstracts (K ≥ 0.8). Of these, 31 were excluded as they were either not original studies or did not examine factors that influence adherence to antiretroviral therapy. The remaining 84 studies were included in our analysis (see Figure 1). There was perfect agreement on the final studies selected between BR and PW. All studies were published in English.

Thirty-seven of the studies were qualitative (see Tables 1 and 2). Twelve used focus groups (total number of patients, *n =* 415) [18–29], 15 used semi-structured interviews (*n =* 729) [30–44], and nine used open-ended questioning (*n =* 694) [45–53] to explore barriers and facilitators to adherence. One study employed a writing intervention to solicit barriers and motivators to adherence [54]. The 47 remaining studies employed a quantitative methodology (surveys) and used structured questionnaires or structured interviews (total *n =* 12,902 [55]) [4,56–100] to determine potential factors. Table 3 displays the quality criteria results for the quantitative studies. No studies reported following up with non-responders to the surveys. Of the total sample of eligible studies, 72 were conducted in developed countries [4,18–25,30–39,44–46,48–50,53–56,58,59,61,62,64–67,69–76,79–81,83,84,86,87,108], and 12 in developing nations [47,52,57,60,63,68,77,78,82,85,94,96]. Fifty-six were from the United States [4,18–26,28,30–36,38–40,46,49–51,53,54,58,59,61,62,66,67,70,71,73,74,76,79–81,84,86,88–91,93,95,108], three from Canada [27,45,72], three from the United Kingdom [55,69,98], two from Italy [56,64], two from France [75,92], two from The Netherlands [42,83], and one each from Australia [48], Switerland [37], and Belgium [44]. Two studies were multinational [65,87]. The studies conducted in developing countries included four from Brazil [47,68,78,85], and one each from Uganda [57], Cote d'Ivoire [63], South Africa [82], Malawi [96], Botswana [52], Costa Rica [94], Romania [60], and China [77]. Tables 4 and 5 outline the factors affecting HAART adherence reported by HIV-positive individuals from developed and developing countries as determined by the qualitative studies.

**Table 5:**
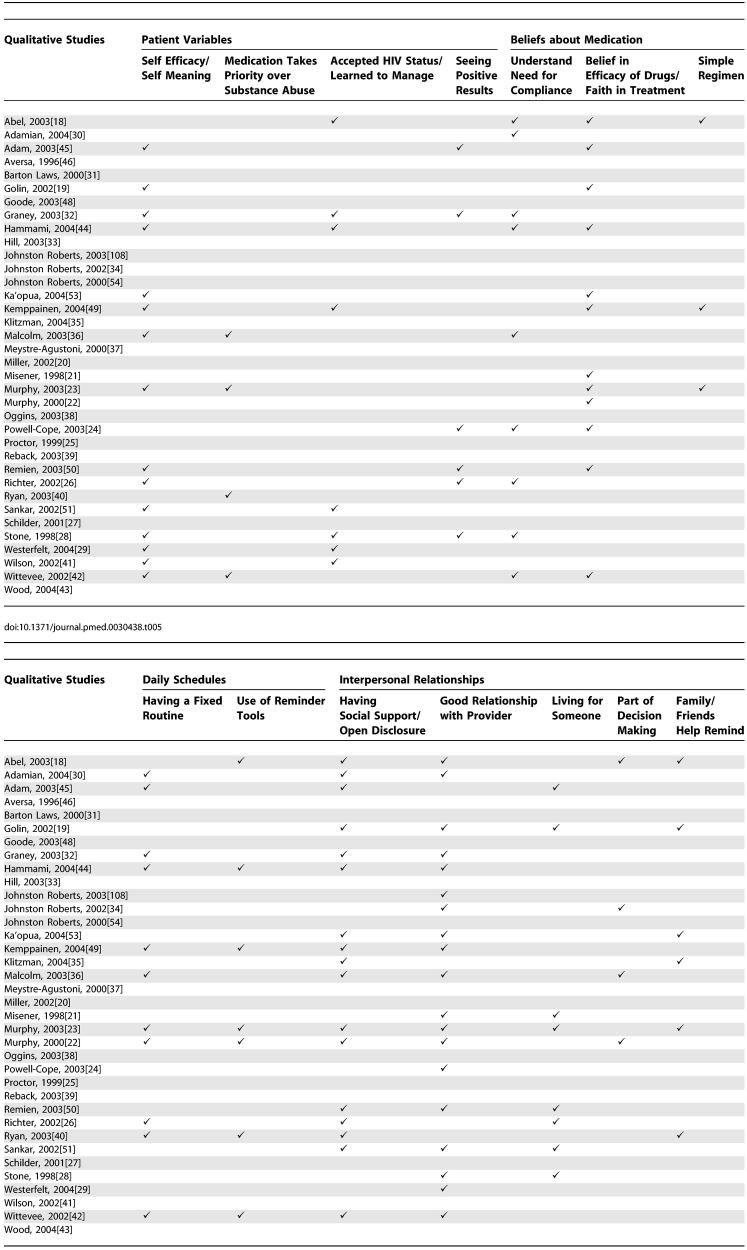
Facilitators Reported in Qualitative Studies

### Barriers and Facilitators Listed by Patients in Developed Countries: Themes from Qualitative Studies 

#### Barriers.

Thirty-three individual themes of barriers were recorded in 34 qualitative studies (see Table 4).

Patient-related: Thirteen barriers were patient-related and included: a fear of disclosure and wanting to avoid taking medications in public places (23/34) [18–20,22–25,27–29,31–33,35–37,40,42,44,45,49–51,108]; feeling depressed, hopeless, or overwhelmed (18/34) [19,23–26,29,31,33,36,40,41,43,45,46,49,50]; having a concurrent addiction (14/34) [23,24,27,31,33,36,39–42,49–51,81]; and forgetting to take medication at the specified time (11/34) [20,24,25,28,31–33,37,40,44,50]. Other barriers include: being suspicious of treatment/medical establishment (9/34) [21,26,35,36,38,41,42,50,51]; wanting to be free of medications or preferring a natural approach (10/34) [20,21,29,31,32,37,44,50,54,108]; feeling that treatment is a reminder of HIV status (8/34) [18,32,38,39,41,43,49,54]; wanting to be in control (7/34) [28,31,37,38,41,54,108]; not understanding treatment instructions (5/34) [31,33,36,38,42]; still having doubt or not being able to accept HIV status (5/34) [18,33,42,44,51]; and a lack of self-worth (4/34) [35,43,44,51]. Financial constraints [31,42,46], being homeless [40,42], and having other concurrent illnesses affecting adherence were also cited.

Beliefs about medication: There were eight reported barriers pertaining to beliefs/perceptions about medications. Some common barriers in this category included: side effects (either real or anticipated) (27/34) [18,20,21,23–32,35,37,38,41–46,48–50,54,108]; complicated regimens (12/34) [18,22,23,26–28,32,42,48–50,54]; and the taste, size, dosing frequency, and/or pill count (12/34) [18,20,23–25,29,45,48–50,54]. In nine studies, when individuals prescribed HAART felt healthy, adherence was often negatively affected [22,24,25,29,32,33,38,43,44]. Other barriers included: doubting the efficacy of HAART (7/34) [21,23,25,26,42,45,46]; having a decreased quality of life (6/34) [20,24,25,38,42,46]; uncertainty of long-term effects (6/34) [30,32,45,46,48,49]; and unwanted changes in body image (5/34) [18,28,37,45,54].

Daily schedules: Nine common barriers were related to daily schedules and included: disruptions in routine or having a chaotic schedule (16/34) [19,22,23,25,27,30,37,39–45,54,108]; finding HAART too inconvenient or difficult to incorporate (14/34) [19,20,27–29,31,32,37,38,41,44,46,48,54,108]; and difficulties coordinating adherence with work, family, or care-giving responsibilities (11/34) [18,20,24,27,28,31,32,37,45,54]. Individuals in seven studies found it difficult to balance the numerous strict dietary requirements associated with HAART [18,19,22,25,30,39,45]. Six studies cited sleeping through a dose [19,29,31,39,40,49]. Other barriers included: being away from home and not bringing medication (6/34) [24,31,33,39,40,42]; being too distracted or busy (5/34) [24,29,33,40,51]; and having no time to refill prescriptions, or other pharmacy-related problems (4/34) [22,24,25,31]. Finally, four studies described difficulties with a particular dose, particularly the middle-of-day or early-morning dose [19,29,42,48].

Interpersonal relationships: Interpersonal relationships can affect adherence behaviors. Twelve studies noted a lack of trust or a dislike of a patient's health-care provider as an impediment to adherence [21–24,27,31,34,36,38,42,49,50]. Ten studies noted social isolation [23,25,33,36,42,44,48–51]. Nine studies noted negative publicity regarding HAART or the medical establishment [21,28,35,36,38,44–46,51]. Finally, five studies noted that having a discouraging social network often deterred patients from successful adherence (5/34) [21,23,28,35,45].

#### Facilitators.

Patient-related: Fourteen factors facilitating successful adherence to HAART were abstracted. Patient-related facilitators included having self-worth (15/23) [19,23,26,28,29,32,36,41,42,44,45,49–51,53], medication taking priority over substance use (4/23) [23,36,40,42] and seeing positive results when adhering to HAART (6/23) [24,26,28,32,45,50]. Also, those patients who had accepted their HIV-seropositivity reported improved adherence (8/23) [18,28,29,32,41,44,49,51].

Beliefs about medication: The most common motivator (12/23) to adherence is a belief in the efficacy of HAART and “having faith” in the treatment [18,19,21–24,42,44,45,49,50,53]. Other motivators included understanding the need for strict compliance (9/23) [18,24,26,28,30,32,36,42,44], and having a simple regimen (3/23)[18,21,49].

Daily schedules: Twelve studies reported learning to balance HAART with daily schedules as a facilitator of adherence. Having a routine in which taking antiretrovirals could be easily incorporated (11/23) [22,23,26,30,32,36,40,42,44,45,49], and making use of reminder tools (7/23) [18,22,23,40,42,44,49] are both reported to be effective tools for optimizing adherence.

Interpersonal relationships: Positive interpersonal relationships were reported as necessary for successful adherence. Having a trusting relationship with a health-care provider was reported as a facilitator of adherence in 17 studies [18,19,21–24,28,29,32,34,36,42,44,45,49–51,53,108]. In addition, openly disclosing HIV status to family and friends and having a strong support network was reported as influential to adherence (18/23) [18,19,22,23,26,30,32,35,36,40,42–45,49–51,53]. Other motivators included: living for someone, especially, children (9/23) [19,21,23,26,28,43,45,50,51]; being actively involved in treatment decision making (4/23) [18,22,34,36]; and using friends and family as reminders (6/23) [18,19,23,35,40,53].

#### Common themes from surveys and quantitative studies.

Figure 2 displays the pooled results of studies assessing barriers and reporting proportions of responders. Table 6 displays the surveys that did inquire of the issues addressed in the qualitative studies. There were three barriers described in qualitative reports but not in the quantitative studies. These were: having suspicions regarding HAART, wanting to be in control, and doubting or having difficulty accepting one's HIV status.

**Table 6:**
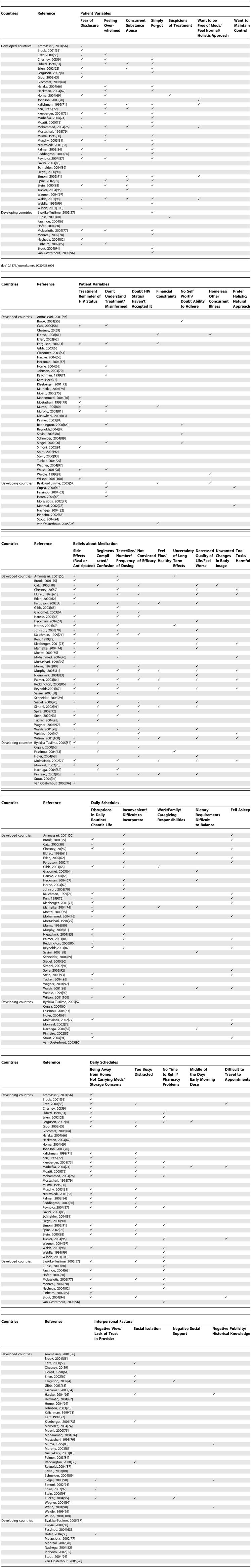
Barriers Reported in Quantitative Studies (Surveys)

Eight quantitative studies reported facilitators to adherence (see Table 7). Four themes for facilitation of adherence were mentioned in the qualitative studies that were not discussed in the relevant quantitative studies (i.e., having medication take priority over substance abuse, having a simple regimen, using reminder tools, and living for someone).

### Barriers Listed by Patients in Developing Countries: Themes from Qualitative Studies

As there were only two studies identified, we describe the findings here. Eighteen specific barriers are cited in two studies [47,52].

Patient-related: The most common patient-related barriers were: having a co-existing substance addiction, simply forgetting, and financial constraints [47,52]. Other barriers affecting adherence incorporated: a fear of disclosure [52]; difficulty understanding both treatment instructions; the need for compliance [47]; and the presence of concurrent diseases or illnesses, including malnutrition [52].

Beliefs about medication: Barriers reflective of patient beliefs regarding antiretrovirals included: side effects (either real or anticipated) [52]; complicated regimens [52]; the taste, size, and frequency of dosing [52]; having doubts about HAART efficacy [47]; feeling fine or healthy [52]; a decreased quality of life while taking medications, or feeling too sick [52]; and being uncertain about potential long-term effects of HIV treatment [47].

Daily schedules: Trouble incorporating work and family responsibilities with HAART was seen as a barrier to adherence in both studies. Traveling long distances to receive treatment was common, and not surprisingly, transportation difficulties were often reported to be a major hindrance to adherence (2/2). Other barriers included running out of medications or having an irregular supply [52]; being away from home [52]; and being too busy or distracted to properly comply [52].

No studies mentioned interpersonal relationships as a barrier to adherence in this population.

No facilitators to adherence were discussed in any study in a developing nation setting.

#### Themes from surveys and quantitative studies.

Ten surveys were found in developing settings (see Figure 3). No quantitative study enquired of difficulties with morning or afternoon doses, work and family responsibilities, or listed inconvenience as a barrier.

## Discussion

To our knowledge, this is the first systematic review to examine the concerns of HIV patients to maintaining adherence. We found that fear of disclosure, forgetfulness, a lack of understanding of treatment benefits, complicated regimens, and being away from their medications were consistent barriers to adherence across developed and developing nations. More common to developing settings were issues of access, including financial constraints and a disruption in access to medications. While there is a tremendous paucity of qualitative research in developing settings, our findings indicate that many barriers to adherence can be addressed with patients through discussion and education regarding treatment benefits to health. In developing settings, access to medications is the greatest concern. Indeed, discussion in both economic settings may alleviate patients' suspicions regarding treatment and address practical barriers to improve adherence. This study should also be used to guide the development of interventions aiming to improve adherence in any setting.

This study has several important strengths. The methods we employed to tabulate these findings come from a multi-step process. We first systematically identified qualitative and quantitative studies examining the questions. We then extracted the themes from the qualitative studies and determined which of them were sampled in the quantitative studies. Finally, we synthesized the available quantitative data. By systematically determining the existence and prevalence of barriers in multiple qualitative and quantitative studies, we believe that stronger inferences can be made into patient-related adherence obstacles and facilitators. We have previously demonstrated that surveys benefit from systematically examining qualitative studies, as this improves content validity [13,101]. To this end, our review of qualitative studies identified several key themes addressing barriers to adherence that were not examined in larger quantitative studies. The presence of barriers in more than one qualitative study, consisting of populations of patients representing different patient populations, supports the conclusion that these barriers are somewhat applicable. Our meta-analysis of survey data is a relatively new process that we have previously demonstrated [102,103], and can permit stronger inferences into the generalizability of our findings. Finally, our criteria to assess the quality of both qualitative studies and surveys are a new contribution to the methodological literature. Recognizing that the absence of reporting particular methodological criteria may not reflect what was actually conducted during a study [104], we invite discussion regarding the relative usefulness and applicability of these criteria.

This work has several limitations. We aimed to reduce reviewer bias by conducting abstraction independently, in duplicate. We cannot, however, know to what extent we may miss themes or to what extent reporting bias of the original report may have contributed. We emphasize that our methodology is specific but not sensitive for identifying themes. Reporting bias in the included manuscripts may have limited our ability to identify all barriers and facilitators to adherence. A broad range of economic and social conditions fall under the Human Development Index. It would wrong to assume that all individuals living in a HDI-categorized “developed” nation are in a better economic situation than all individuals living in a “developing” nation. Detailed information pertaining to this was rarely available in the original reports included in this review. It is possible that surveys used in developing nations were similar to surveys used in developed nations. However, the validity of these surveys in developing settings may not be appropriate, and we press for further qualitative research on this topic. Detailed population descriptions (e.g., education level) and the regional conditions from which this study is produced (e.g., gross national product) would benefit interpretation of future studies in this field. There are several interpretations of appropriate adherence and execution of drug regimens. We did not evaluate patients' perceptions of what “adherence” mean to them, whether it meant acceptance, execution, or persistence of drug therapy [105]. In our meta-analyses of pooled survey data, we found large heterogeneity (as displayed by the I^2^ values in Figures 2 and 3), indicating large variation between the surveys. Very little methodological literature deals with pooling proportions, and our findings call for further exploration to determine the importance of this heterogeneity. Finally, there were few studies in developing countries that studied early adopters to antiretroviral therapy. These individuals may not be representative of the larger epidemic and may not have experienced longer-term side effects of therapy.

**Table 7:**
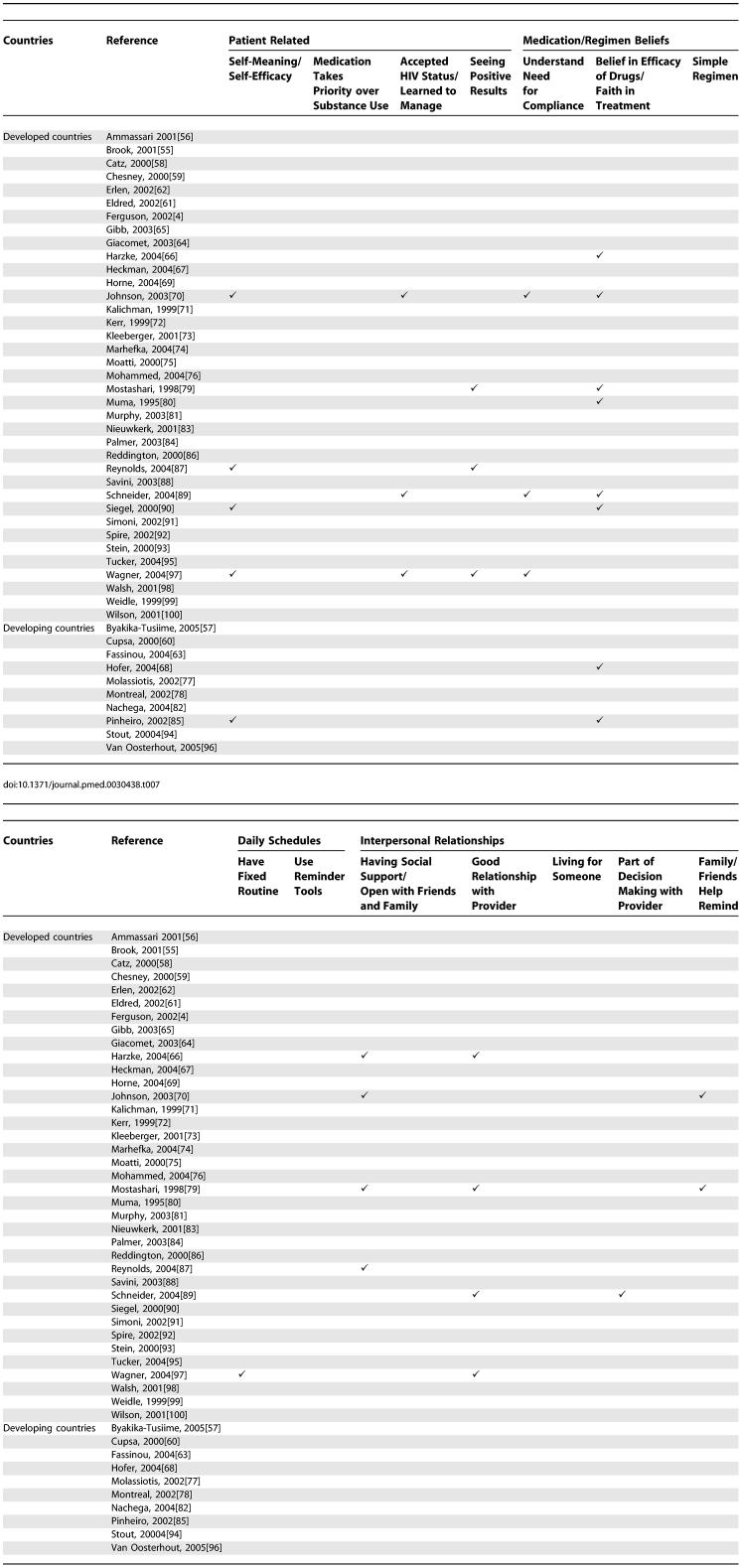
Facilitators Reported in Quantitative Studies (Surveys)

It is important to note that the qualitative studies generated a richer spectrum of barriers and facilitators than did the quantitative studies. Qualitative studies are superior at identifying patient-important barriers and facilitators. We would submit that the ideal study of adherence would be one that occurs across several phases and incorporates both qualitative and quantitative elements. For example, to avoid biasing one's investigation with a priori assumptions about what may be important factors relating to adherence in a given population, it is logical to commence a study with qualitative research, thereby allowing the local population to tell the researchers what they believe to be important barriers, rather than the reverse. By using questionnaires developed in settings that are economically or culturally foreseeably different, the surveys force respondents to answer potentially irrelevant questions.

Clearly, the evidence base for barriers and facilitators of adherence is far richer from developed countries than from developing countries. In our analysis we found only two qualitative studies published from developing nation settings. This is sadly paradoxical, given that the vast majority of HIV/AIDS patients live in the developing world, and over the coming decades will constitute a growing proportion, and probably the majority, of the world's HAART recipients. Consequently, we see further research on HAART adherence in developing countries that incorporates both qualitative and quantitative elements as a priority.

**Figure 3:**
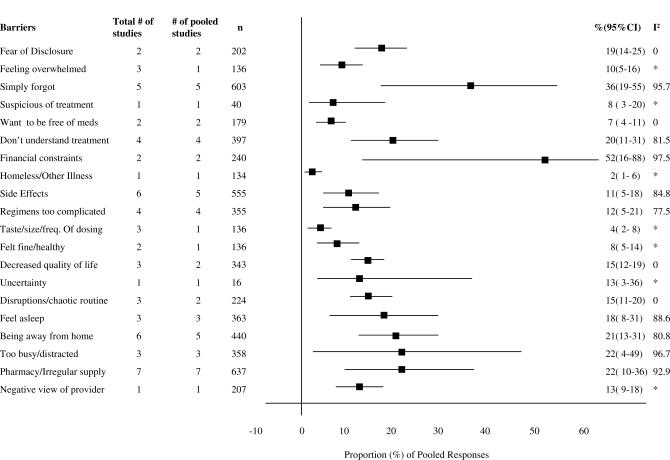
Barriers Reported in Developing Countries

Our findings should influence adherence program delivery systems in developing settings. We found that issues such as fear of disclosure, suspicions about treatment, forgetfulness, and irregular supply were important barriers identified by large proportions of the populations studied. It seems appropriate that before mandating any adherence program, such as disclosure or accompagnateurs, opportunities should be provided for individuals who require opting out [106,107]. Further, in developing settings, the reliability of medication access is an important adherence barrier that individuals have little opportunity to facilitate. Patient-level adherence can be determined only when a steady supply of medication exists.

We identified a broad range of barriers and facilitators to adherence. These barriers should be inferred as guides for interventional research to improve adherence rates. Given the many factors tabulated in this review, clinicians should use this information to engage in open discussion with patients to promote adherence and identify barriers and facilitators within their own populations. The methodology we used to pool the quantitative data is novel and may prove a useful methodological tool for generalizing patient-important issues.

## Abbreviations

HAART: highly active antiretroviral therapy
